# Comprehensive Analytical Studies on the Solubility and Dissolution Rate Enhancement of Tadalafil with Type IV Lipid Formulations

**DOI:** 10.3390/pharmaceutics17111436

**Published:** 2025-11-07

**Authors:** Günay Husuzade, Burcu Demiralp, Hakan Nazlı, Tuğçe Boran, Sevgi Güngör

**Affiliations:** 1 Institute of Graduate Studies in Health Sciences, Istanbul University, Istanbul 34116, Türkiye; husuzade.gunay@gmail.com; 2 Department of Pharmaceutical Technology, Faculty of Pharmacy, Istanbul University, Istanbul 34116, Türkiye; bmesut@istanbul.edu.tr; 3 Department of Pharmaceutical Technology, Faculty of Pharmacy, Trakya University, Edirne 22030, Türkiye; hakannazli@trakya.edu.tr; 4 Department of Pharmaceutical Toxicology, Faculty of Pharmacy, Istanbul-Cerrahpaşa University, Istanbul 34320, Türkiye; tugce.boran@iuc.edu.tr

**Keywords:** Tadalafil, Type IV lipid-based formulations, low solubility, Caco 2 cell line, analytical studies, lipolysis

## Abstract

**Background**: This work aimed to enhance the solubility of Tadalafil (TDL), a BCS Class II drug, by preparing Type IV lipid-based formulations. **Methods**: Type IV formulations were prepared using surfactants and/or hydrophilic co-surfactants, resulting in oil-free systems. **Results**: Based on the solubility test, Transcutol^®^ HP exhibited the highest solubility for TDL (48.33 ± 0.004 mg/mL) and was selected as the co-surfactant. Among surfactants, Kolliphor^®^ PS80 (42.74 ± 2.29 mg/mL), Kolliphor^®^ EL (41.87 ± 2.50 mg/mL), Kollisolv^®^ PEG 400 (40.70 ± 0.30 mg/mL), and Kolliphor^®^ HS15 (31.40 ± 3.63 mg/mL) demonstrated high solubilization capacity. These were used to prepare formulations without the addition of an oil phase. The developed formulations resulted in a system with a nano-droplet size (<50 nm) and PDI values < 0.3, which was clear, transparent, and resistant to pH dilutions. The optimum Type IV lipid formulations were further characterized and demonstrated good thermodynamic stability under temperature and pH changes. The optimized formulation was adsorbed onto different carriers and transformed into solid TDL-loaded formulations. The in vitro dissolution rate of the drug from the solidified lipid formulations was studied in various dissolution media. It was observed that the solid formulations prepared with Neusilin US2^®^ (2:1) exhibited a significantly higher dissolution of over 95% within 5 min compared to the marketed product. The in vitro lipolysis studies demonstrated that F2 formulation maintained TDL in a supersaturated state throughout digestion, with limited enzymatic degradation of the excipients. Cytotoxicity evaluation using the MTT assay in Caco-2 cells confirmed the biocompatibility of both drug-free and TDL-loaded formulations, with IC_50_ values of 19.55 µg/mL and 17.55 µg/mL, respectively. **Conclusions**: The overall results suggested that the developed solid Type IV lipid formulations can improve the dissolution rate of TDL, which would potentially lead to an improvement in its oral bioavailability and, consequently, a reduction in the treatment dose as a safe delivery system.

## 1. Introduction

Tadalafil is a phosphodiesterase-5 (PDE-5) inhibitor drug for oral erectile dysfunction (ED) use [1], which was approved by the US Food and Drug Administration (FDA) in November 2003 [2]. It is currently available in different doses (2.5 mg, 5 mg, and 20 mg) as a film-coated tablet [3]. Tadalafil is a moderately lipophilic (log P: 2.48 [4]) with low solubility (in water: 0.3 mg/L) compound [5]. Based on the Biopharmaceutical Classification System (BCS), it is classified as a Class II (low solubility, high permeability) drug [5,6]. The poor aqueous solubility is one of the significant drawbacks of Tadalafil [7], leading to low oral bioavailability. Thus, different strategies were used to enhance its solubility and, consequently, oral bioavailability [7,8].

Physically, lipid-based formulations (LBFs) form various systems such as emulsions, self-emulsifying drug delivery systems (SEDDSs), self-microemulsifying drug delivery systems (SMEDDSs), self-nanoemulsifying drug delivery systems (SNEDDSs), micellar solutions, and lipid solutions [9]. Recently, it has been noticed that in comparison with other technologies, lipid-based formulations are commonly used in order to improve the solubility and then the bioavailability of poorly soluble drugs [10]. There are some drugs on the market that were developed using LBFs, such as Sandimmune^®^ and Neoral^®^ (cyclosporine A), Kaletra^®^ (lopinavir and ritonavir) [11], Amitiza^®^ (lubiprostone), Rocaltrol^®^ (calcitriol), and Lipofen^®^ (fenofibrate) [12,13].

LBFs, classified from a very oily solution to an aqueous micellar solution, were proposed in different subgroups by Pouton in 2006. They were categorized according to their compositions into four types, involving Type I, II, IIIA, IIIB, and IV formulations [14]. Type I formulations are simple formulations and only contain triglyceride or mixed glyceride lipids (100% lipids). Type II formulations comprise glyceride lipids (40–80%) and water-insoluble (lipophilic) surfactants (20–60%). Type III formulations are prepared with glyceride lipids, a water-soluble (hydrophilic) surfactant, and a co-surfactant. Type III formulations are divided into two subgroups, Type IIIA and Type IIIB, based on the amount of ingredients. Type IIIA formulations include a higher proportion of glyceride lipids and a lower proportion of surfactant and co-surfactant (40–80%, 20–40%, and 0–20%, respectively), while Type IIIB formulations contain limited amounts of glyceride lipids (less than 20%) and a greater amount of surfactant (20–50%) and a co-surfactant (20–50%). Type IV formulations are simply composed of a surfactant (water-soluble (0–20%) or water-insoluble (30–80%)) and a co-surfactant (0–50%) [15,16].

LBFs maintain the drug in a dissolved state during its passage through the gastrointestinal tract [10,17]. Specifically, Type IV formulations consist exclusively of hydrophilic surfactants and/or hydrophilic cosolvents, making them oil-free systems [11]. These formulations initially exhibit high solvent capacity and form aqueous micellar solutions [15]. Such micellar solutions are generated when surfactants are used above their critical micelle concentration (CMC) and with a hydrophilic–lipophilic balance (HLB) above 12, allowing drug solubilization in aqueous media at low concentrations [18]. As noted by Fatouros and Mullertz, absorption takes place during the mixed micellar phase, where drug solubilization occurs in the gastrointestinal tract [9]. Moreover, micellar solubilization can enhance luminal solubility by up to 1000-fold [19]. Type IV formulations are particularly suitable for lipophobic and hydrophobic drugs (insoluble in both water and oil) [10,11]. A well-known marketed example of a Type IV formulation is Amprenavir (Agenerase^®^ capsules, GSK, UK) [20]. Since most LBF excipients are in liquid form, additional techniques are needed to convert these liquid formulations into solid dosage forms (e.g., capsules or tablets) [21,22].

Solidification of liquid lipid formulations addresses limitations of capsule liquid-filling, such as chemical instability, capsule leakage, incompatibility, and higher manufacturing costs [23]. Several techniques, including adsorption onto solid carriers/porous adsorbents, freeze drying, spray drying, hot-melt extrusion, and spin evaporation, have been applied to obtain solid LBFs [14,15,24,25]. Among these, adsorption onto micronized adsorbents is considered the simplest and most practical method. In this approach, liquid formulations are mixed with an adsorbent in a blender, and the resulting powder can be directly encapsulated or further processed into tablets with additional excipients [23,26]. The choice of adsorbent depends on properties such as surface area, particle size, pore diameter, and oil adsorption capacity. Commonly used adsorbents for solidifying LBFs include Aerosil, Hubersorb, Sylysia, Syloid, Florite, and Neusilin [23,27,28].

The purpose of this study was to improve the solubility of TDL, a BCS Class II drug substance, by optimization of Type IV LBFs and then to solidify the selected TDL-loaded LBFs with different types of adsorbents. The developed formulations in a solid powder form were assessed by in vitro dissolution studies, and the dissolution rate performance of the optimized solid Type IV LBFs was compared to the marketed product of TDL.

## 2. Materials and Methods

### 2.1. Materials

TDL was a gift from Jubilant Life Sciences (Noida, Uttar Pradesh, India). Transcutol HP, Labrasol, Gelucire 44/14, and Gelucire 48/16 were kind gifts from Gattefossé (Saint-Priest, France). Kolliphor PS 20, Kolliphor PS 60, Kolliphor PS 80, Kolliphor HS 15, Kolliphor PEG 400, Kolliphor CS 12, Kolliphor CS 20, Kolliphor EL, and Kolliphor ELP were obtained as a gift from BASF (Istanbul, Turkey). Adsorbent powders were received as gifts from suppliers, including Florite R (Tomita Pharmaceutical Co., Ltd., Tokyo, Japan), Neusilin US2 and UFL2 (Fuji Chemical, Tokyo, Japan), and Syloid XDP 3050 and 3150 (Grace GmbH, Worms, Germany). Bile salt (B3883), pancreatic extract (P1625), Tris maleate, and 4-bromophenylboronic acid (B75956) were purchased from Sigma-Aldrich (Steinheim, Germany). Phospholipid (Lipoid S100) was purchased from Lipoid (Ludwigshafen, Germany). Calcium chloride dihydrate, and sodium chloride were purchased from Merck (Darmstadt, Germany). All the other used chemical materials were of analytical grade and high-performance liquid chromatography (HPLC) grade.

### 2.2. Analytical Studies

Based on Turkish Pharmacopeia-II (European Pharmacopoeia 9th edition 2017), analysis of TDL was conducted by the HPLC (Shimadzu LC 20-AT, Kyoto, Japan) method under specific conditions, which involved photodiode array detector, wavelength (285 nm), a mobile phase consisting of buffer (1 mL of trifluoroacetic acid mixed and diluted to 1000 mL with purified water) and acetonitrile at a ratio of 55:45 *w*/*w*, AcclaimTM 120 C18 column (4.6 × 150 mm, 5 μm, Thermo Scientific^TM^, Waltham, MA, USA), column temperature (40 °C), and flow rate (1.5 mL/min) [29]. The analytical method was validated according to ICH Q2.

### 2.3. Solubility Studies

The equilibrium solubility of TDL in different excipients (surfactants and co-surfactants) was determined using the shaking method [10]. A total of 1 mL of each excipient was taken to the Eppendorf (Isolab, Laborgeräte GmbH, Eschau, Germany), adding an excess amount of TDL, and then vortexed (IKA^®^ Vortex-Genius 3, IKA^®^-Werke GmbH & CO.KG, Staufen, Germany). Afterward, the sealed Eppendorf was shaken at 37.00 ± 0.05 °C, 100 rpm for 48 h via an orbital shaker (Model 420, Thermo Electron Corporation^®^, Waltham, MA, USA). When the time finished, each mixture was centrifuged at 15,000 rpm for 15 min (Model D-7200, Hettich, Kirchlengern, Germany). In a volumetric flask, a certain amount of supernatant was taken and diluted with mobile phase (in Section 2.6). After filtration through a 0.45 μm membrane filter (25 mm × 0.45 μm Nylon, Alwsci^®^, Shaoxing, China) and analyzed in triplicate by HPLC, as described in Section 2.2.

### 2.4. Preformulation Studies

According to the solubility data of TDL, its Type IV LBF was prepared with surfactant and co-surfactants, which provided the highest solubility of TDL. Surfactant and co-surfactant mixtures (S_mix_) in different proportions (1:1, 1:2, and 2:1 *w*/*w*) [17] were mixed using a magnetic stirrer (IKA^®^-RT15 Power, IKA^®^-Werke GmbH & CO.KG, Germany) and then used. Clear-transparent formulations without phase separation were selected and then characterized by the following methods. Type IV formulations without TDL were also prepared.

The formulation details are given in Table 1.

### 2.5. Characterization and Evaluation of Type IV Formulations

#### 2.5.1. Droplet Size and Polydispersity Index (PDI) Analysis

The droplet size and PDI of both TDL-unloaded and -loaded Type IV LBFs were measured by dilution of distilled water (Merck Milli-Q, Darmstadt, Germany) and analyzed with the ZetaSizer instrument (1000 HS, Malvern Instruments, Worcestershire, UK). Measurements were carried out at 37.00 ± 0.05 °C and an angle of 90° during 60 s of equilibration. Each sample was measured repeatedly 3 times with 12–17 runs. Formulations possessing droplet sizes below 100 nm and PDI 0.3, respectively, were selected, and further characterization studies were conducted [30,31].

#### 2.5.2. Transmittance Determination (T, %)

Transmittance values (%) were measured using a UV–Vis Spectrophotometer (UV 1601, Shimadzu, Japan) device at 650 nm wavelength after diluting each TDL-unloaded Type IV LBFs with purified water 100 times. The test was repeated three times, and purified water was used as a blank. Formulations having a high percentage of transmittance were chosen [32].

#### 2.5.3. Robustness to Dilution

Dilution of formulations at different physiological pHs has a crucial effect on the phase separation of emulsifying systems [33,34]. According to the literature, the impact of dilution in solutions that mimic the pH of the physiological fluids of the gastrointestinal tract (GIT) was examined. This test was carried out by diluting TDL-unloaded Type IV LBFs into various pH-buffered solutions (1.2, 4.5, 6.8, and 7.4) and kept at room temperature for 48 h. The droplet sizes and PDI values were measured, as described in Section 2.5.1.

### 2.6. Preparation of TDL-Loaded Type IV Formulations

According to the characterization studies performed on the TDL-unloaded Type IV formulations, the most physically stable formulations were selected. A total of 2 mL of the selected formulations (S_mix_ mixture) was added to an Eppendorf tube, and then 40 mg of TDL was added and vortexed (IKA^®^ Vortex-Genius 3, IKA^®^-Werke GmbH & CO.KG, Germany). The TDL-loaded Type IV LBFs were shaken at 37.00 ± 0.05 °C and 100 rpm by an orbital shaker (Model D-7200, Hettich, Germany) for 48 h. Then, each Type IV LBF was centrifuged (Model D-7200, Hettich, Germany) at 15,000 rpm for 15 min and analyzed, as mentioned in Section 2.2.

### 2.7. Thermodynamic Stability Studies

Based on the data obtained, the selected TDL-loaded Type IV formulations were evaluated in different conditions to find their resistance to changes that may be faced due to external factors. The droplet size, PDI values, and phase separation were assessed for each formulation as briefly explained above [35]. Thermodynamic stability studies regarding the effect of different pHs (1.2, 4.5, 6.8, and 7.4) and temperatures (−20 ± 2 °C and 25 ± 2 °C) were implemented on the selected TDL-loaded Type IV LBFs.

The selected formulations were diluted 20-fold with purified water. The diluted samples were centrifuged at 15,000 rpm for 30 min and then stored for 2 days at low temperature (−20 ± 2 °C) and room temperature (25 ± 2 °C) [36,37]. The test was repeated at least 3 times. The physical stability of the TDL-loaded Type IV LBFs was evaluated visually based on their phase separation and precipitation, as well as the droplet size and PDI measurements, as shown in Section 2.5.1.

The stability of the TDL-loaded Type IV formulations was analyzed in triplicate under different pH buffer solutions (1.2, 4.5, 6.8, and 7.4). The phase separation and precipitation of Type IV LBFs were assessed visually, and the droplet size and PDI values of them were measured, as explained in Section 2.5.1.

### 2.8. Preparation of Solid TDL-Loaded Type IV Lipid Formulations

LBFs are fabricated as solid carriers using porous adsorbents to overcome their obstacles, such as emulsion aggregation, phase separation, and relatively rapid degradation of the active ingredient, which can be displayed in the liquid form [38]. Adsorbent capacity, flowability, and not leaving an oily feeling are the most important parameters in adsorbent selection [39]. According to the results of the study conducted, as described in Section 2.5.1, the TDL-loaded Type IV lipid formulations with droplet size < 100 nm and PDI value < 0.3 were selected. This liquid form was converted to a solid powder form by screening the suitable adsorbents, namely, Florite R, Neusilin US2, Neusilin UFL2, Syloid XDP 3050, and Syloid XDP 3150.

The oil adsorption capacity was measured for each solid carrier, defined as the amount of adsorbent required to convert the unit dose of the oily formulation to a solid free-flowing powder [40]. Each adsorbent was placed separately in the porcelain capsule, and the selected TDL-loaded Type IV lipid formulations were added dropwise until a non-sticky, free-flowing powder was obtained.

### 2.9. Dissolution Studies

The dissolution rate of TDL from the solidified Type IV formulation (F1–F10) was compared with the reference product, Cialis^®^ 5 mg (Eli Lilly and Company, Indianapolis, IN, USA). The in vitro dissolution rate test was performed using the USP apparatus II paddle (Sotax, Aesch, Switzerland) method at 50 rpm. The used medium was 900 mL of 0.1 N HCl (pH = 1.2) solution at 37.00 ± 0.05 °C. The samples (3 mL) were taken at different time intervals (1, 3, 5, 10, 15, 20, 30, and 45 min). Directly, an equal volume of fresh medium was exchanged to keep the sink conditions [41,42]. In addition, the formulations that showed high dissolution rates were studied in different dissolution media (1000 mL), including buffer solutions with pH 4.5 and 6.8 and containing 0.5% sodium lauryl sulfate (SLS) recommended by the FDA [42]. Each experiment was performed in triplicate. Then, each sample was filtered through a 0.45 μm membrane filter (25 mm × 0.45 μm Nylon, Alwsci^®^, China).

### 2.10. In Vitro Lipolysis Test

Tris-maleate (2 mM), calcium chloride dihydrate (1.4 mM), sodium chloride (50 mM), bile salt (2.95 mM), and phospholipid (0.26 mM) containing medium was mixed with 300 µL Type IV formulation in a thermostatically controlled vessel (37.00 ± 0.5 °C) for 15 min. The pH of the medium was adjusted to 6.5 with 0.4 M NaOH solution. The pancreatic extract was freshly prepared just before the test. Briefly, 1 g of pancreatin powder was added to a 5 mL digestion buffer (free of bile salt and phospholipid) and mixed for 15 min. Then, the suspension was centrifuged at 4000 rpm (2075× *g*). The enzyme solution obtained was added to the lipolysis medium to initiate digestion. Since there are two stages here, the samples were withdrawn at 1, 5, 10, and 15 min after the dispersion phase started and 5, 15, 30, 45, and 60 min after the digestion phase started. At specified times, quantities of 1 mL samples were withdrawn, and the lipase activity was inhibited by the addition of 5 µL 4-BBBA solution in methanol. The samples were centrifuged at 13,500 rpm (12,225× *g*) for 15 min. TDL in the supernatant was quantified by HPLC using the validated method.

### 2.11. Cell Culture and Cytotoxicity Assay

The Caco2 (Human colorectal adenocarcinoma, HTB-37) cell line was purchased from American Type Culture Collection (ATCC, USA) and was grown in 20% fetal bovine serum (FBS) and 1% penicillin/streptomycin/amphotericin containing Dulbecco’s Modified Eagle Medium: Nutrient Mixture F-12 (DMEM-F12) medium. The cell culture medium was changed every 2–3 days. Sub-culturing was performed when the cells reached 60–70% confluency.

Cell viability was measured using the MTT (3-(4,5-dimethylthiazol-2-yl)-2,5-diphenyl-2H-tetrazolium bromide) assay. The cells were counted using trypan blue dye for the evaluation of cell viability, and then 1 × 10^4^ cells were seeded into each well in a flat-bottomed 96-well plate. After cellular attachment, the cells were exposed to 50–4.56 μg/mL TDL containing the F2-TDL formulation and the same volume of drug-free F2 formulation. The cells were treated with different concentrations of formulations for 24 h. A total of 20 µL of MTT dye solution was added to each well and incubated at 37.00 ± 0.5 °C for 3 h. In mitochondria-active cells yellow-colored MTT dye was metabolized to purple-colored formazan crystals. Formazan crystals were dissolved in 100 µL DMSO, and absorbance was read at 590 nm in a microplate reader (Epoch, Nuremberg, Germany). Cell viability was calculated as a percentage of the control group. The experiments were performed in triplicate.

## 3. Results

### 3.1. Analytical Studies

**Figure 1:**
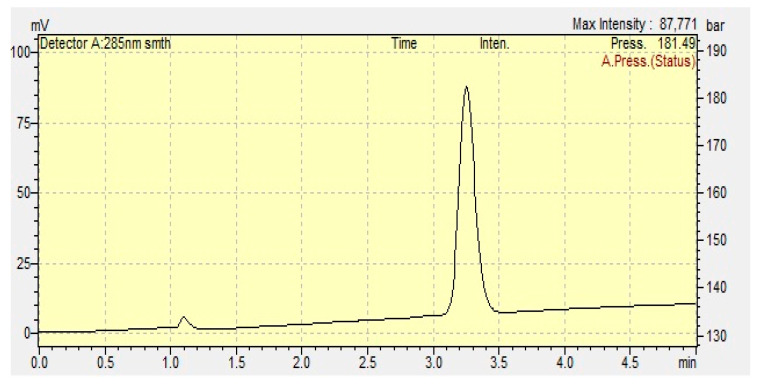
The TDL chromatogram.

According to the analytical method validation studies conducted in accordance with the ICH Q2(R1) guidelines, TDL demonstrated a well-defined and sharp chromatographic peak with a retention time of approximately 3.0 min, as shown in Figure 1. The calibration curve, constructed using seven concentrations ranging from 0.5 to 6.0 µg/mL, exhibited excellent linearity, with the regression equation determined to be y = 1 × 10^8^x − 7024.8 and a correlation coefficient (r^2^) of 0.999, indicating strong linearity across the tested range. The method demonstrated acceptable precision, with relative standard deviation values below 2% for both intra-day and inter-day analyses. Accuracy, assessed through recovery studies, yielded mean recovery values within the acceptable range of 98–101%, confirming the reliability of the method for quantitative analysis of TDL.

### 3.2. Solubility Studies

**Figure 2:**
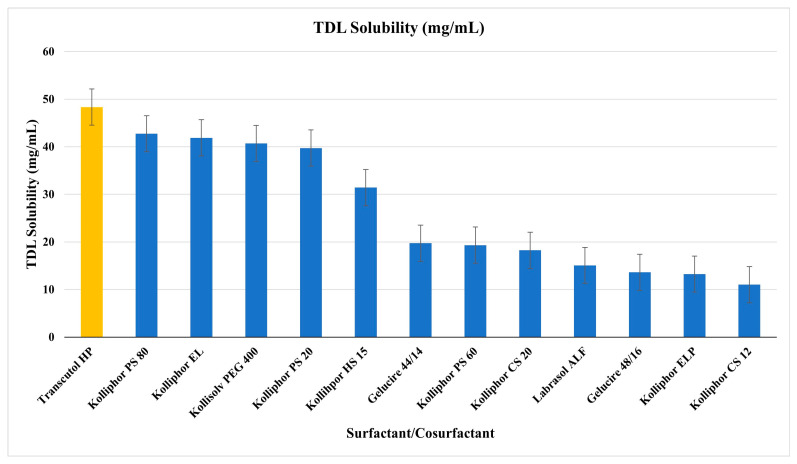
The solubility of TDL in different surfactants (blue bars) and co-surfactants (yellow bars) (mg/mL, mean ± SD, n = 3).

The selection of excipients is critical, and making the correct choice should be based on the solubility capacity and the stability of the excipients. The surfactants and co-surfactants chosen for formulation development were primarily determined by their maximum drug solubilization capacity. As a result of this study, Transcutol HP (48.33 ± 0.004 mg/mL) was selected as the co-surfactant due to its superior solubilization capacity. The following surfactants were selected based on their higher solubilization as well: Kolliphor PS 80 (42.74 ± 2.29 mg/mL), Kolliphor EL (41.87 ± 2.50 mg/mL), Kollisolv PEG 400 (40.70 ± 0.30 mg/mL), Kolliphor PS 20 (39.73 ± 1.07 mg/mL), and Kolliphor HS 15 (31.40 ± 3.63 mg/mL), as shown in Figure 2.

### 3.3. Characterization and Evaluation of TDL-Unloaded Type IV Formulations

#### 3.3.1. Droplet Size and PDI Analysis

Nine S_mix_ mixtures (Khs15T11-KELT21) were prepared in different ratios of 1:1, 1:2, and 2:1 from the selected surfactants and co-surfactants based on the preformulation studies. The droplet size and PDI value were measured in purified water. As shown in Table 2, the droplet size of the prepared formulations is less than 100 nm, and the PDI value is less than 0.3. This is consistent with most of the literature [43,44,45,46].

#### 3.3.2. Transmittance Determination (T, %)

The mixture consisted of the diluted Type IV lipid formulations with purified water should be transparent and clear, visually [37]. Khs15T11, KPS80T12, KELT12 and KELT21 formulations produced a clear distribution with 100%, Khs15T12, Khs15T21, KPS80T11, KPS80T21 and KELT11 formulations with 99.77% transmittance values. This means all the formulations (water-soluble surfactants) can form transparent micellar solutions and maintain the drug in soluble form [10].

#### 3.3.3. Robustness to Dilution

Based on the resulting droplet size and PDI value, only three formulations (Khs15T21, KPS80T21, and KELT21) among the nine formulations were not significantly impacted by the change in the pH of the medium, as presented in Table 3. It indicates that these formulations have the ability to form stable solutions (no aggregation) when diluted in gastrointestinal fluids [47,48]. However, there was a significant change in the Khs15T21 formulation at pH 7.4 after 48 h.

#### 3.3.4. Preparation of TDL-Loaded Type IV Formulations

The solubility study of TDL was implemented in the chosen three formulations. The Khs15T21, KPS80T21, and KELT21 formulations were 29.94 ± 0.46 mg/mL, 24.88 ± 1.14 mg/mL, and 22.66 ± 0.46 mg/mL, respectively. These results were obtained using a vortex and orbital shaker, even though formulations were visually observed to contain small drug particles (TDL). Moreover, it has been noticed that 2 mL of the formulation can completely dissolve 15 mg of TDL without using any techniques. Accordingly, the best and most soluble amount of TDL in the 1 mL formulation was determined to be 7.5 mg.

#### 3.3.5. Thermodynamic Stability Studies

Khs15T21, KPS80T21, and KELT21, TDL-loaded, exhibited thermodynamic stability under centrifuge, cold, and ambient conditions. The thermodynamic stability data showed no signs of aggregation, precipitation, turbidity, or phase separation of the formulations. This was supported by studies related to the thermodynamic stability of micellar solutions by other researchers [49,50,51]. In addition, KPS80T21 and KELT21 TDL-loaded LBFs were found to be resistant to different pH buffer solutions (1.2, 4.5, 6.8, and 7.4). Table 4 and Table 5 indicate that the droplet sizes and PDI values of 7.5 mg TDL-loaded LBFs are less than 100 nm and 0.3, respectively. These results emphasize that any physical changes in the formulation were not observed.

### 3.4. Solidification of the Selected LBFs

The solid carriers used (Florite R, Neusilin US2, Neusilin UFL2, Syloid XDP 3050, and Syloid XDP 3150) to solidify the LBFs prepared are considered safe (GRAS), and they are used effectively to produce solid Type IV lipid formulations [52,53]. The maximum amount of Type IV lipid formulation is adsorbed on the minimum amount of solid carrier to ensure a free-flowing powder. The adsorbents used in this study are shown in Table 5.

The KPS80T21 and KELT21 TDL-loaded formulations were solidified with five different types of adsorbents for further studies. According to the properties of the adsorbent, Florite R, Neusilin US2, and Neusilin UFL2 have a high loading capacity [54,55], while Syloid XDP 3050 and Syloid XDP 3150 showed a low loading capacity [56]. The percentage of each formulation is displayed in Table 6.

### 3.5. Dissolution Studies

**Figure 3:**
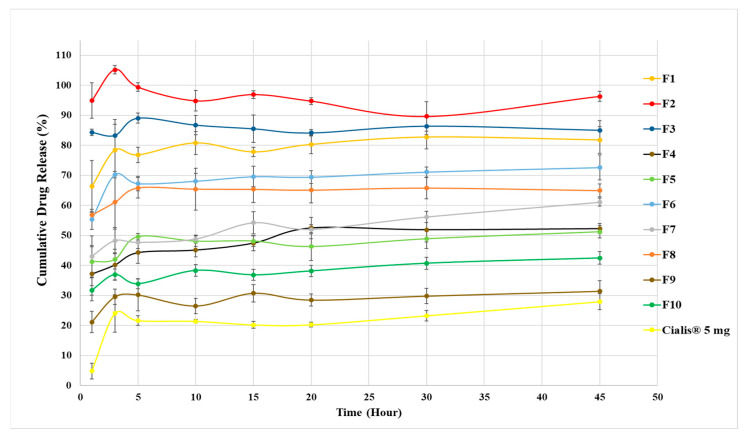
The dissolution profile of formulations and marketed product in a dissolution medium (pH 1.2) (mean ± SD, n = 3).

**Figure 4:**
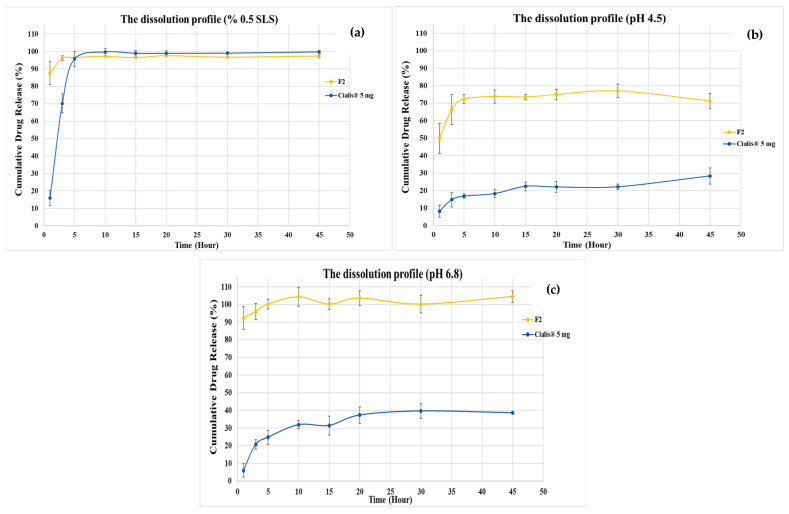
The dissolution profile of formulation F2 and the marketed product: (**a**) 0.5% SLS in purified water dissolution medium, (**b**) pH 4.5 buffer solution dissolution medium, (**c**) pH 6.8 buffer solution dissolution medium (mean ± SD, n = 3).

The solid TDL-loaded Type IV LBFs (F1–F10) in powder form were evaluated in terms of their in vitro drug release pattern compared with the marketed product (Cialis^®^ 5 mg, Eli Lilly and Company, Madrid, Spain). The drug release behavior of TDL in dissolution medium mimicking gastric media (pH 1.2) is presented in Figure 3. The solid TDL-loaded Type IV LBFs presented an enhancement in TDL release compared to the marketed formulation. The F2 (KPS80T21) formulation showed the highest drug release with 96% released TDL within 45 min. Therefore, dissolution profiles of the F2 formulation and marketed product were examined in different dissolution media (pH 1.2, pH 4.5, and pH 6.8), which mimic gastrointestinal transit media, to evaluate their in vitro performance. The release rate of TDL from solid TDL-loaded Type IV lipid formulation was significantly enhanced, as exhibited in Figure 4a–c. Usage of SLD for dissolution studies increased the release of TDL. However, the marketed product presented an improvement in dissolution medium with 0.5% SLS. Type IV lipid formulations can effectively improve the drug dissolution rate and oral bioavailability of BCS Class II drugs [10].

### 3.6. In Vitro Lipolysis Test Result

**Figure 5:**
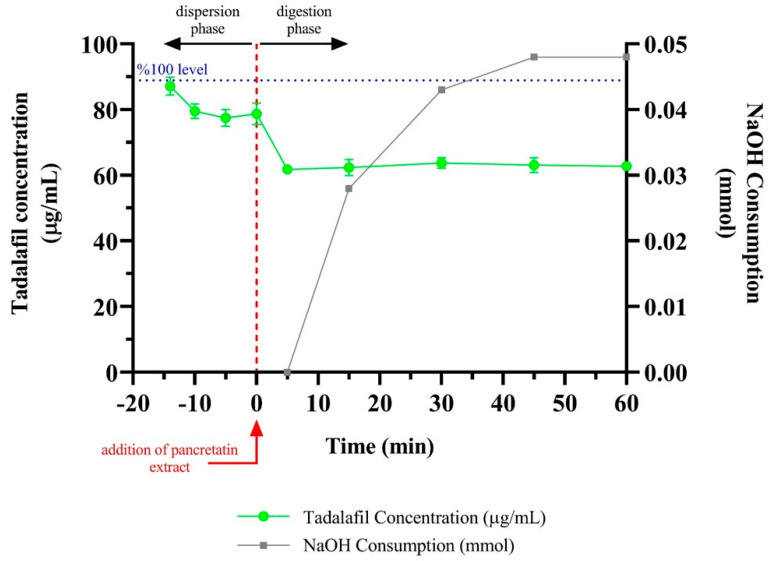
The in vitro lipolysis of the F2 formulation.

The concentration of TDL in the aqueous solution during the in vitro lipolysis test of the F2 formulation is shown in Figure 5.

In the lipolysis experiment, 0.3 mL formulation, which equals 2.22 mg TDL, was added to the lipolysis medium. Therefore, 100% TDL concentration in dispersion and digestion steps was approximately 89 µg/mL and 74 µg/mL, respectively. The rate of in vitro lipolysis depends on the lipid-based drug delivery system. Usually, the lipolysis rate decreases from type I to type IV systems due to decreased lipidic content [57]. F2 formulation can be considered a type IV formulation since it consists of Kolliphor^®^ PS80 as a surfactant and Transcutol^®^ HP as a co-surfactant. An aqueous phase and a pellet formed in the sample tubes after separation in the centrifuge. The decrease in the quantified TDL amount in the aqueous phase was observed as soon as the test started. This observation was due to the loss of solvent capacity with the dilution of the formulation. Approximately a 10% decrease (from 87 µg/mL to 79 µg/mL) in the concentration of dissolved TDL was seen in the dispersion phase. Despite the reduction, TDL concentration was still well above the supersaturated state. The rapid decrease in TDL concentration after the digestion phase was mainly due to the added pancreatic extract increasing the volume of the lipolysis medium. In the digestion phase, TDL concentration remained constant since the digestion of excipients was quite limited. Kolliphor^®^ PS80 was only marginally digested, and Transcutol^®^ HP was undigestible [58]. Therefore, excipients were potentially still available for the solubilization of TDL during lipolysis. The volume of NaOH consumed during the lipolysis experiment was also very low, as digestion was limited.

### 3.7. Cell Culture and Cytotoxicity Assay

**Figure 6:**
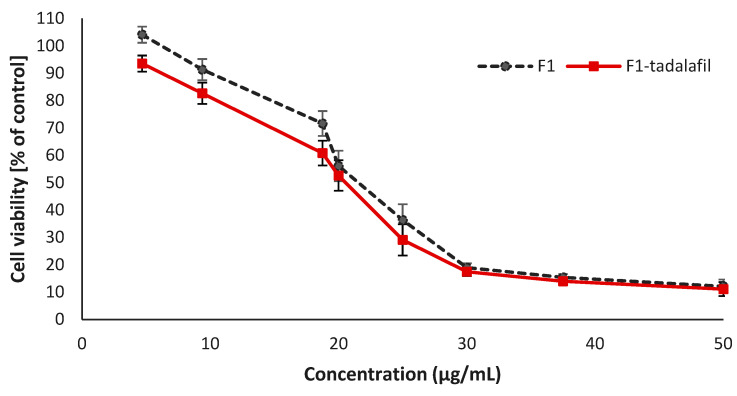
Cytotoxicity profiles of F2 and F2-TDL (mean ± SD, n = 3).

In the literature, it was observed that the C_max_ concentration of TDL was calculated as 0.378 µg/mL at the 2nd hour after a single oral dose of 20 mg TDL intake [59]. In this study, the half Maximal Inhibitory Concentration (IC_50_) of formulations was determined as 17.55 µg/mL for the F2-TDL-loaded formulation. To account for any carrier-related effects, cells were additionally treated with the same volume of the equivalent drug-free F2 formulation for each drug concentration examined. Drug-free F2 formulation and F2-TDL-loaded formulation exhibited similar toxicity to Caco2 cells, as seen in Figure 6. When all data are compared, it appears to be safe.

## 4. Discussion

The development of LBFs represents a promising strategy for enhancing the solubility and oral bioavailability of poorly water-soluble drugs, particularly those classified as BCS Class II compounds. In the present study, TDL was incorporated into Type IV LBFs using different combinations of surfactants and co-surfactants, and the performance of these formulations was systematically evaluated in terms of solubility, droplet size, stability, dissolution, in vitro lipolysis, and cytotoxicity. The findings highlight not only the critical role of excipient selection but also the importance of physicochemical stability in determining the ultimate in vitro performance of the formulations.

The choice of excipients is a pivotal factor in the successful design of LBFs, as surfactants and co-surfactants directly influence solubilization capacity, dispersion properties, and stability of the formulation. In this study, Transcutol HP demonstrated the highest solubilization capacity (48.33 ± 0.00 mg/mL), which is consistent with its well-documented ability as a hydrophilic co-surfactant to improve drug solubilization [41]. Among surfactants, Kolliphor PS 80, Kolliphor EL, and Kolliphor HS 15 showed superior solubilization capacities compared to the other evaluated excipients. These results are in line with previous studies emphasizing the role of non-ionic surfactants, particularly polyethylene glycol (PEG)-based derivatives, in enhancing solubility and drug loading in LBFs [41]. The observed differences in solubilization between excipients emphasize that the rational selection of surfactants is crucial for maximizing drug incorporation and ensuring formulation efficiency.

Droplet size and PDI are two critical parameters influencing the absorption and stability of lipid-based formulations. The developed S_mix_ combinations produced droplet sizes below 100 nm and PDI values below 0.3, which are generally considered favorable for oral absorption [41,42,43]. The small droplet size provides a large interfacial surface area, thereby facilitating drug release and absorption in the gastrointestinal tract [44,45]. The current results are consistent with previous reports where LBFs with nanosized droplets demonstrated improved absorption of BCS Class II drugs [46]. Moreover, the uniform distribution reflected in the low PDI values suggests physical stability of the formulations, which is essential for maintaining drug solubilization during storage and after dilution.

The robustness to dilution further confirmed the stability of selected formulations under gastrointestinal conditions. Among the tested formulations, Khs15T21, KPS80T21, and KELT21 maintained stable droplet sizes and PDI values across different pH media, indicating their suitability for oral delivery [47,48]. However, instability was observed in the Khs15T21 formulation at pH 7.4 after 48 h, with significant increases in droplet size and PDI. This finding suggests that while certain surfactant–co-surfactant combinations are effective in acidic and near-neutral environments, their stability may be compromised in more basic conditions, possibly due to structural rearrangements or aggregation. Such observations underline the importance of conducting extended stability testing under physiological conditions to predict in vivo behavior more accurately.

Stress condition tests, including storage at −20 ± 2 °C and 25 ± 2 °C, demonstrated that the selected formulations maintained their physical stability without visible aggregation, precipitation, turbidity, or phase separation. These results support the physical robustness of the micellar systems and are consistent with prior reports describing the colloidal stability of Type IV LBFs [49,50,51]. Although the observed results suggest promising stability under the tested conditions, the limited temperature range does not allow for a comprehensive evaluation of thermodynamic stability in the strictest regulatory sense. Further studies under ICH-recommended long-term and accelerated storage conditions would be necessary to confirm this. Nonetheless, the consistent performance of the TDL-loaded KPS80T21 and KELT21 formulations across a range of pH buffers reinforces their suitability for oral delivery, where dynamic gastrointestinal environments must be tolerated.

The dissolution performance of the solidified (powder) formulations provided particularly compelling evidence of the superiority of Type IV LBFs compared to conventional oral dosage forms. All the powdered TDL-loaded LBFs (F1–F10) demonstrated improved drug release compared to the marketed Cialis^®^ tablet, with the F2 formulation showing the most rapid and extensive release, reaching 96% within 45 min. The dramatic improvement over the reference product highlights the ability of LBFs to overcome the dissolution-limited absorption associated with tadalafil and other BCS Class II drugs. Notably, even in different dissolution media (pH 1.2, 4.5, and 6.8), F2 consistently outperformed the marketed product, which required surfactant (0.5% SLS) to achieve comparable release. These results corroborate earlier findings where lipid-based systems were shown to significantly enhance dissolution rate and oral bioavailability of poorly soluble drugs [10].

The in vitro lipolysis test provided valuable mechanistic insight into the performance of the optimized F2 formulation. A decrease in TDL concentration during the dispersion phase was observed, primarily due to dilution effects and reduced solvent capacity. However, the concentration of TDL remained above the supersaturation threshold, suggesting that the formulation effectively maintained the drug in a solubilized state. Supersaturation is a key determinant of absorption for BCS Class II drugs, as it prolongs the duration of drug availability for intestinal uptake [5]. This observation also raises the possibility that the apparent solubilization may partially reflect the presence of TDL in dispersed nanosized aggregates, rather than purely molecularly dissolved forms, given their potential to pass through filtration membranes used during sample processing. The limited digestion observed in the in vitro lipolysis study of the F2 formulation can be attributed primarily to its formulation as a Type IV lipid-based system, which does not contain digestible triglycerides. This characteristic is further supported by the formulation’s excipient composition Kolliphor^®^ PS80, a surfactant with low susceptibility to enzymatic hydrolysis, and Transcutol^®^ HP, a non-digestible co-surfactant. Consequently, the formulation preserved sufficient solubilization capacity throughout the digestion phase [58]. This finding is particularly relevant, as premature drug precipitation during digestion has been reported as a major challenge for some lipid-based systems. The ability of the F2 formulation to maintain supersaturation suggests a high likelihood of improved in vivo performance.

Cytotoxicity assays further confirmed the safety of the developed formulations. The TDL-loaded F2 formulation exhibited IC_50_ values (17.55 µg/mL) that were significantly higher than C_max_ (0.378 ng/mL) of TDL 2 h after 20 mg oral administration [59]. This indicates a wide safety margin and supports the potential of the formulation for oral administration without introducing additional cytotoxic risks. The comparable cytotoxicity profiles of drug-free and drug-loaded formulations also suggest that the excipients themselves, at the tested concentrations, are well tolerated by intestinal epithelial cells.

Taken together, these findings highlight the significant potential of solidified Type IV LBFs for improving the solubility, dissolution, and oral bioavailability of tadalafil. The enhanced dissolution behavior, stability under gastrointestinal conditions, maintenance of supersaturation during lipolysis, and favorable cytotoxicity profile all suggest that the developed formulations could offer superior therapeutic performance compared to conventional tadalafil tablets. Beyond tadalafil, the results indicate that this formulation strategy may be broadly applicable to other BCS Class II drugs, particularly where dissolution is the primary barrier to absorption. Future in vivo pharmacokinetic studies are warranted to confirm the translational relevance of these in vitro findings and to further establish the clinical potential of the developed formulations.

## 5. Conclusions

In this study, a new TDL-loaded Type IV LBFs consisting of Kolliphor PS80 as a surfactant and Transcutol HP as a co-surfactant was developed. The optimum formulation of Type IV lipid structure was further characterized and provided good thermodynamic stability. The developed formulations were a system with a small droplet size of less than 50 nm, clear, transparent, and resistant to pH dilutions. Then, the selected formulation (F2) was adsorbed on different carriers (Florite R, Neusilin US2, Neusilin UFL2, Syloid XDP 3050, and Syloid XDP 3150) and transformed into solid TDL-loaded Type IV lipid formulations. It has been shown that the Type IV lipid formulations with Neusilin US2 increase the dissolution rate of TDL. Thereby, the developed lipid solid carrier system of TDL is expected to have a rapid absorption rate compared to the conventional oral preparation in tablet form, which would result in increasing the bioavailability of the drug, and consequently reducing the treatment dose. In order to confirm the data obtained in this study, it is recommended to conduct future pre-clinical animal and bioavailability studies.

## Figures and Tables

**Table 1:** The codes and compositions of surfactant and co-surfactant mixtures (S_mix_) and their ratios used in lipid-based formulations.

Codes *	Surfactant/Co-Surfactant	Ratios (*w*/*w*)
Khs15T11 *	Kolliphor HS 15^®^/Transcutol HP^®^	1:1
Khs15T12 *	Kolliphor HS 15^®^/Transcutol HP^®^	1:2
Khs15T21 *	Kolliphor HS 15^®^/Transcutol HP^®^	2:1
KPS80T11 *	Kolliphor PS 80^®^/Transcutol HP^®^	1:1
KPS80T12 *	Kolliphor PS 80^®^/Transcutol HP^®^	1:2
KPS80T21 *	Kolliphor PS 80^®^/Transcutol HP^®^	2:1
KELT11 *	Kolliphor EL^®^/Transcutol HP^®^	1:1
KELT12 *	Kolliphor EL^®^/Transcutol HP^®^	1:2
KELT21 *	Kolliphor EL^®^/Transcutol HP^®^	2:1

* Kolliphor HS 15^®^, Khs15; Kolliphor PS80^®^, KPS80; Kolliphor EL^®^, KEL; and Transcutol HP^®^, it is expressed with T abbreviations.

**Table 2:** Droplet size and PDI value of Type IV lipid formulations *.

Formulations (S_mix_) and Codes	Droplet Size	PDI **
Kolliphor HS 15/Transcutol (1:1)—**Khs15T11**	37.74 ± 36.26	0.14 ± 0.04
Kolliphor HS 15/Transcutol (1:2)—**Khs15T12**	91.24 ± 32.01	0.15 ± 0.04
Kolliphor HS 15/Transcutol (2:1)—**Khs15T21**	12.91 ± 0.19	0.15 ± 0.00
Kolliphor PS 80/Transcutol (1:1)—**KPS80T11**	62.02 ± 39.30	0.24 ± 0.11
Kolliphor PS 80/Transcutol (1:2)—**KPS80T12**	60.27 ± 37.48	0.13 ± 0.04
Kolliphor PS 80/Transcutol (2:1)—**KPS80T21**	48.06 ± 3.83	0.17 ± 0.05
Kolliphor EL/Transcutol (1:1)—**KELT11**	15.77 ± 3.94	0.11 ± 0.05
Kolliphor EL/Transcutol (1:2)—**KELT12**	13.71 ± 0.23	0.15 ± 0.05
Kolliphor EL/Transcutol (2:1)—**KELT21**	26.56 ± 12.43	0.12 ± 0.04

* (nm, mean ± SD, n = 3), ** (mean ± SD, n = 3).

**Table 3:** Droplet size and PDI value of TDL-unloaded Type IV lipid formulations at different pH media.

Formulations	pH = 1.2		pH = 4.5		pH = 6.8		pH = 7.4	
	Droplet Size *	PDI **	Droplet Size *	PDI **	Droplet Size *	PDI **	Droplet Size *	PDI **
Khs15T21	14.03 ± 1.69	0.17 ± 0.03	27.87 ± 8.40	0.11 ± 0.06	12.85 ± 0.15	0.07 ± 0.01	15.01 ± 2.48	0.17 ± 0.05
KPS80T21	43.03 ± 4.62	0.16 ± 0.11	76.28 ± 34.54	0.25 ± 0.07	34.51± 40.03	0.18 ± 0.05	39.79 ± 44.34	0.24 ± 0.15
KELT21	15.00 ± 1.83	0.17 ± 0.04	14.10 ± 0.03	0.08 ± 0.03	13.70 ± 0.21	0.16 ± 0.01	14.06 ± 0.17	0.16 ± 0.03
48 h ***								
Khs15T21	31.43 ± 30.14	0.19 ± 0.03	34.76 ± 35.99	0.23 ± 0.00	12.78 ± 0.13	0.16 ± 0.02	5504.00 ± 82.78	0.73 ± 0.23
KPS80T21	17.89 ± 4.87	0.15 ± 0.07	190.90 ± 53.09	0.30 ± 0.17	69.27 ± 17.57	0.23 ± 0.08	177.60 ± 90.20	0.29 ± 0.17
KELT21	14.69 ± 0.17	0.10 ± 0.02	71.23 ± 51.53	0.22 ± 0.07	99.22 ± 16.10	0.22 ± 0.10	47.38 ± 32.40	0.15 ± 0.06

* (nm, mean ± SD, n = 3), ** (mean ± SD, n = 3). *** measurements were repeated at the end of 48 h.

**Table 4:** Droplet size and PDI values of TDL-loaded Type IV lipid formulations in purified water.

Formulations (S_mix_)	Droplet Size *	PDI **
KPS80T21	32.99 ± 26.85	0.12 ± 0.04
KELT21	13.71 ± 0.67	0.11 ± 0.01

* (nm, mean ± SD, n = 3), ** (mean ± SD, n = 3).

**Table 5:** Resistance of TDL-loaded Type IV lipid formulations to pH dilutions.

Formulations (S_mix_)	pH = 1.2		pH = 4.5		pH = 6.8		pH = 7.4	
	Droplet Size *	PDI **	Droplet Size *	PDI **	Droplet Size *	PDI **	Droplet Size *	PDI **
KPS80T21	12.61 ± 2.72	0.16 ± 0.05	28.97 ± 13.90	0.18 ± 0.03	140.50 ± 9.47	0.17 ± 0.01	29.50 ± 14.54	0.16 ± 0.03
KELT21	13.80 ± 0.16	0.06 ± 0.01	22.17 ± 14.16	0.16 ± 0.04	14.59 ± 1.96	0.12 ± 0.02	14.03 ± 0.13	0.11 ± 0.06

* (nm, mean ± SD, n = 3), ** (mean ± SD, n = 3).

**Table 6:** Composition of solid Type IV formulations.

Formulations (S_mix_)	TDL Amount (mg)	S_mix_: Adsorbent Ratio (*w*/*w*)	Type of Carrier	Solid TDL-Loaded Type IV Formulations
KPS80T21	7.5	2:1	Florite R	F1
KELT21				F6
KPS80T21	7.5	2:1	Neusilin US2	F2
KELT21				F7
KPS80T21	7.5	2:1	Neusilin UFL2	F3
KELT21				F8
KPS80T21	7.5	1.5:1	Syloid XDP 3050	F4
KELT21				F9
KPS80T21	7.5	1.5:1	Syloid XDP 3150	F5
KELT21				F10

## Data Availability

The original contributions presented in this study are included in the article. Further inquiries can be directed to the corresponding author.
